# Efficacy and safety of alpha lipoic acid-capped silver nanoparticles for oral applications†

**DOI:** 10.1039/c9ra00613c

**Published:** 2019-02-28

**Authors:** G. C. Cotton, C. Gee, A. Jude, W. J. Duncan, D. Abdelmoneim, D. E. Coates

**Affiliations:** Faculty of Dentistry, Sir John Walsh Research Institute, University of Otago Dunedin New Zealand gemma.cotton@otago.ac.nz

## Abstract

Silver nanoparticles (AgNPs) are widely studied for their broad-spectrum antimicrobial effects, and can be utilised readily in biomaterials, however the cellular safety of specific AgNP formulations should be profiled prior to clinical usage. This study determined the cytotoxic effect of small sized (6 nm) alpha lipoic acid capped-AgNPs on human gingival fibroblasts (HGF), as compared to ionic silver and clinical antiseptics. The metabolic pathway was investigated to determine the cellular effects on HGF cells. The minimal inhibitory concentration (MIC) and minimal bactericidal concentration (MBC) was established for a range of oral related bacteria. Results showed that cell viability decreased with increasing AgNP concentration, whereas lower concentrations of AgNPs, (≤5 μg ml^−1^) caused a significant increase in cell proliferation at 24 and 72 hour time points. The cytotoxicity profile of AgNPs exhibited significantly lower concentrations, relative to the dose of clinical efficacy, when compared to clinical antiseptics. Caspase 3/7 was not significantly altered when HGF cells were treated with 0.225 μg ml^−1^ AgNPs, indicating cell necrosis rather than apoptosis. Quantitative RT^2^-PCR detected an upregulation of genes associated with oxidative stress and the G2M cell cycle checkpoint at ≤4 hours, but expression levels returned to levels consistent with control cells at 24–96 hours. An MIC range of 2.5–12.5 μg ml^−1^ (min. *Escherichia coli*, *Streptococcus mutans*, *S. mitis*; max. *Staphylococcus aureus*) was determined across the bacterial species tested and an MBC range of 5–100 μg ml^−1^ (min. *E. coli*, max. *S. mutans*). The antimicrobial profile was similar to that of AgNO_3_ which suggested that the antimicrobial effect may be influenced by free Ag^+^ release. It was concluded that alpha lipoic acid capped AgNPs possess limited cytotoxic activity to HGF cells when compared to clinically utilised oral antiseptics, observed *via* the cellular recovery after initial AgNP treatment and a lack of cumulative cytotoxic effect, whilst maintaining a broad range antimicrobial effect of the AgNPs.

## Abstract 2

Presentation of the limited cumulative cytotoxic effects of an alpha lipoic-acid capped silver nanoparticle on human gingival fibroblasts, with broad range antimicrobial activity, for potential use in biomaterials.

## Graphical abstract

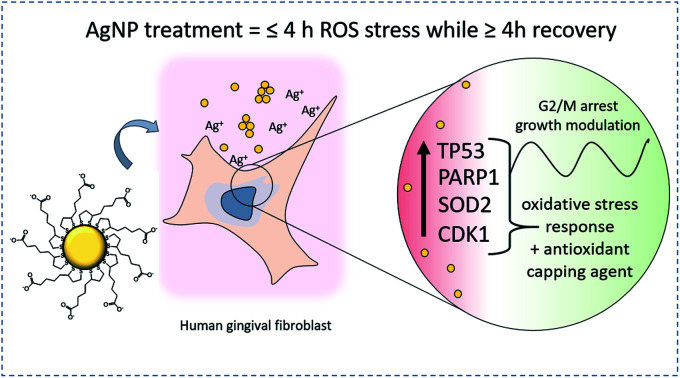

## Introduction

The use of nanoparticles, 1–100 nm in any given dimension, has had a significant impact in the field of materials science due to their high surface area-to-mass ratio compared to the corresponding bulk material. In the case of silver nanoparticles (AgNPs), the phenomena of enhanced broad-spectrum antimicrobial activity has been well established and reported.^1–5^ AgNPs are a promising alternative antimicrobial agent that are increasingly being used in clinically applied biomaterials to inhibit microbial colonisation and subsequent infection.^6–9^ They are already clinically used in wound dressings, catheters and implants, for prophylactic and/or therapeutic treatment.^10^

The resulting antimicrobial properties of AgNPs have been attributed to a variety of physiochemical characteristics including size, shape, and surface functionalisation/capping agent.^2,11,12^ AgNPs as a large family of products, have differing mechanisms whereby they exert their antimicrobial action and varying efficacy as determined by their minimal inhibition/bactericidal concentration (MIC/MBC). Furthering this concept, it is likely that the toxicological effects of AgNPs on human cells may be dependent on the different manufacturing technologies used to produce particles with unique physiochemical properties. In the case of AgNPs the capping agent and particle size are known to alter NP-cell interactions, aggregation profiles, and free Ag^+^ ion release – all known variables that affect cytotoxicity.^13–16^

It has previously been shown that the AgNP capping agent influences the cytotoxic behavior of the nanoparticles^17,18^ and it is also generally considered that cytotoxicity increases with smaller sized particles and in a dose- and time-dependent manner.^19^ The AgNPs synthesized and assayed within this research are 1–12 nm in diameter, a relatively small sized particle when compared to those studied previously.^20^ It has also been reported that AgNPs can result in the induction of oxidative damage and inflammatory lesions in human gingival fibroblast cells.^21^ The particles using in this research are capped with alpha lipoic acid, a five-membered cyclic disulphide tailing a short hydrocarbon chain on one end and a carboxylic group on the other. Alpha lipoic acid is known to act as an antioxidant and be anti-inflammatory.^22–24^ A literature study suggested that AgNPs capped with alpha lipoic acid demonstrate a reduced toxicity when compared to other capping agents including, uncapped, and polyethylene glycol particles.^17^

As with any new therapeutic, it is essential to investigate not only the targeted efficacy but the safety profile of lipoic acid AgNP on contacted cells in a clinical scenario. Primary human cells are the gold standard for *in vitro* testing of new therapeutics and in this study primary human derived gingival fibroblasts were used for examining the potential of lipoic acid AgNP for intra-oral topical application and as a representative *in vitro* system.^18^ With this in mind, the present study investigated the antimicrobial effects of alpha lipoic acid-capped AgNPs on a range of oral bacteria and the cytotoxic effects of these AgNPs on primary human gingival fibroblasts. The cytotoxicity of silver nitrate (an ionic silver comparison), chlorhexidine and silver diamine fluoride (two clinical controls) were investigated for comparative purposes. We also report insight into the mechanistic and molecular effects of the AgNPs on apoptosis and cell cycle regulation. The use of these nanoparticles is part of an ongoing investigation into creating regenerative biomaterials that deter bacterial infection at the site of implantation. The project aimed to examine the cellular and molecular impact of AgNPs when engineered to have a small size to enhance its antimicrobial action, and with a capping agent to reduce oxidative cytotoxicity.

## Materials and methods

### Silver nanoparticle synthesis

The alpha lipoic acid capped-AgNPs were prepared as described by Dumas *et al.*^25^ To prepare the microemulsions (μEms), 40 ml of an AOT (docusate sodium salt ≥ 96%, Cat. No. 86140, Sigma Aldrich, Missouri, USA) solution in 0.33 M heptane (Cat. No. H350-1, Fischer Scientific, New Hampshire, USA) were placed in two separate flasks. To the first solution, an aqueous solution of silver nitrate (AgNO_3_; Cat. No. 10224350; Fisher Scientific, New Hampshire, USA) (1.6 ml, 0.13 M) was added dropwise with stirring, forming μEm 1. To the second solution, an aqueous solution of sodium borohydride (NaBH_4_ crystalline 98–99%, Cat. No. ICN10289425, Fischer Scientific, New Hampshire, USA) (1.6 ml, 1.84 M) was added dropwise with stirring, forming μEm 2. The flasks were placed in separate ice baths, and μEm 1 was covered with aluminium foil then μEm 2 added dropwise with continuous stirring. Upon addition, a color change from light yellow to dark yellow-brown was observed, suggesting the production of AgNPs. This mixture was allowed to stir in the dark for up to 24 h. Subsequently, alpha lipoic acid (0.08 mM, dissolved in 0.25 ml ethanol) was introduced and the microemulsion was stirred for an additional 2 min. Upon discontinuation of the stirring, a 1 : 1 methanol/acetone mixture was added at an equivalent half volume of the combined microemulsion (40 ml). Phase separation was clearly observed, and a dark-colored interface formed between the two phases, where the particles resided. The nanoparticles at the interface were carefully collected. Subsequently, the particles were washed 3 times with ethanol, with centrifugation at 6000*g* for 5 min, and resuspended in 1–6 ml of deionised (DI) H_2_O (depending on the [Ag] desired) which was previously pre-adjusted to pH 10 (±0.5) with anhydrous ammonia. The resulting yellow-brown colloidal suspension was centrifuged twice at 16 000*g* for 45 min, with the final supernatant collected and retained for characterisation.

### Silver concentration quantification – inductively coupled plasma-mass spectrometry

AgNP-containing samples were prepared for inductively coupled plasma-mass spectrometry (ICP-MS) analysis through the addition of 4 ml of concentrated HNO_3_ in a Teflon digestion vessel (Cat. No. 010-500-264; SPS Science, Quebec, Canada), followed by gentle heating. The samples were then digested at 95 °C for 1 h. During this time, the volume of the digested solution was reduced to ∼0.2 ml, then made up to 3 ml total volume with deionized H_2_O. ICP-MS analysis was performed on an Agilent 7500ce instrument (Agilent Technologies; California, USA) to determine the silver concentration.

### Dynamic light scattering

DLS experiments were performed at 37 °C on a Malvern Zetasizer Nano ZS (Malvern Instruments; Malvern, UK) which used a detection angle of 173°, and a 3 mW He–Ne laser operating at a wavelength of 633 nm. Measurements were performed in triplicate, with each reported value being the average value of 12 runs, and a minimum temperature equilibration time of 120 s was used in all cases. Poly dispersity index (PDI) values were obtained from correlation functions using the Multiple Narrow Modes algorithm based on a non-negative least square fit using Zetasizer software (v. 6.20; Malvern Instruments).

### Transmission electron microscopy

Colloidal AgNP samples were prepared for analysis by depositing 10 μl onto plasma glowed, carbon-coated (400 mesh) copper grids. After 60 s, the excess volume was carefully blotted with filter paper and the sample was allowed to air dry before analysis. Transmission electron microscopy (TEM) images were obtained using a Philips CM100 BioTWIN transmission electron microscope (Philips/FEI Corporation; Eindhoven, Holland) equipped with a LaB6 emitter fitted with a MegaView III Olympus digital camera. The nanoparticle size was determined from TEM images using the particle analysis plugin in conjunction with Image J software.

### Silver nanoparticle aggregation assay

The synthesized AgNPs (22.5 μg ml^−1^) were dispersed in DMEM (DMEM; Cat. No. 10569010, Life Technologies New Zealand Limited, Auckland NZ), containing increasing concentrations of FBS (0–10%) (FBS; Cat. No. 10091148, Life Technologies New Zealand Limited, Auckland NZ). Measurements were performed aseptically at 37 °C using DLS, as previously described, after incubation for 0, 1, 24, and 96 h. In-between measurements the samples were incubated in the dark at 37 °C.

### Bacterial preparation

*Escherichia coli* DH5α (*E. coli*), *Staphylococcus aureus* Oxford NCTC6571 (*S. aureus*), *Pseudomonas aeruginosa* OT15 (*P. aeruginosa*), *Streptococcus mutans* UA159 (*S. mutans*), *Streptococcus gordonii* DL1 (*S. gordonii*) and *Streptococcus mitis* IL8 (*S. mitis*) were obtained from the University of Otago culture collection, Dental School and subcultured on agar plates; *P. aeruginosa*, *S. aureus* and *E. coli* were cultured on Tryptic Soy agar (TSB; Cat. No. 1335, Fort Richard, Mt. Wellington, New Zealand) and incubated at 5% CO_2_, 37 °C for 24 h. All streptococci were subcultured on Columbia Sheep Blood agar (CBA; Cat. No. 1100, Fort Richard, Mt. Wellington, New Zealand) under anaerobic conditions and incubated at 5% CO_2_, 37 °C for 48 h. Prior to experiments, the appropriate broth, tryptic soy broth (TSB; Cat. No. 211825, Difco Laboratories, Detroit, MI, USA) for aerobes and brain heart infusion broth (BHI; Cat. No. 237500, Difco Laboratories, Detroit, MI, USA) for anaerobes, were inoculated with a bacterial culture from agar and incubated at 5% CO_2_, 37 °C for 16 h.

### Bacterial inhibition and bactericidal assays

Minimum inhibitory concentration (MIC) and minimum bactericidal concentration (MBC) assays were performed according to a modified Clinical & Laboratory Standards Institute (CLSI) reference method^26^ with *n* = 4 per test and control condition. Assays were performed in thin walled 0.2 ml PCR tubes and were subsequently transferred to 96-well flat-bottomed microtiter plates (Cat. No. 655180, greiner bio-one, CELLSTAR® 96W Microplate, Sigma, Auckland, NZ) for MIC determination through optical density measurements. Each 0.2 ml tube contained 200 μl of DMEM/10% FBS with doubling concentrations from 0.5–200 μg ml^−1^ of AgNP, free silver as AgNO_3_, or no silver as the control. Each tube contained 10 μl of bacterial culture adjusted to a MacFarland standard of 0.5. The tubes were placed into a holder, and secured within a rotating incubator (5 rpm), in the dark, at 37 °C for 24 h. Samples were then transferred to a 96-well plate and the optical density (Abs_600_) was measured on a Synergy 2 Multi-mode microplate reader (Bio Tek Instruments, Inc., Winooski, VT, USA). Background absorbance was determined by performing the assay with AgNP-containing DMEM/10% FBS with 10 μl of a dead bacterial culture (pre-killed in a water bath at 60 °C for 30 min) adjusted to a MacFarland standard of 0.5. MBC was determined by spot-plating (*n* = 4) 10 μl onto agar plates (TSA or CBA) followed by incubation (as above). MBC was determined as the lowest concentration resulting in complete killing to the test bacterium.

### Cytotoxicity

Primary human gingival fibroblasts (HGF) were expanded from three healthy female patients (aged 30–45 years) undergoing routine crown-lengthening surgery at the Faculty of Density, University of Otago. All experiments were performed in accordance with the guidelines of the National Ethics Advisory Committee, New Zealand. This study was approved by the Human Ethics Committee, University of Otago, reference number H17/112 and all patients provided informed consent prior to the procedure. The fibroblasts were phenotyped as previously described.^27^

Cells were seeded at 6000 cells per cm^2^ in 200 μl of culture medium containing DMEM/10% FBS, 50 μg ml^−1^ of Gentamicin (Cat. No. 15710064; Life Technologies) and 1× of antibiotic–antimycotic (Cat. No. 15240062; Life Technologies Ltd). HGFs (*n* = 3; biological replication) were plated into 96-well microtiter plates (*n* = 3; technical replication) and were incubated for 12 h at 37 °C and 5% CO_2_. The culture medium was then replaced with 100 μl per well of fresh culture medium containing AgNP (0.0225, 0.05, 0.225, 0.5, 2.25, 5, 10, 12.5, 22.5 or 50 μg ml^−1^) or an equivalent volume of the Ag carrier, alkaline deionized H_2_O (pH 10.01), in the control wells and were incubated for 24, 48, 72 or 96 h. The AgNP carrier water (pH 10.01), was tested to assess its effects on the assay at equivalent volumes to that added for 50 μg ml^−1^ (2.92 μl/100 μl) and 22.5 μg ml^−1^ (1.32 μl/100 μl) of AgNPs as well as being compared to wells with no carrier. Clinical controls tested under the same conditions and over the same time points were chlorhexidine digluconate, 0.2%, 0.02%, 0.002%, 0.0002% (CHX; 20% stock solution; Cat. No. C9394-25; Sigma-Aldrich), silver diamine fluoride (SDF, Riva Star; Cat. No. 8800501, SDI, Australia) and silver nitrate, 0.05, 0.5, 5, and 50 μg ml^−1^ (AgNO_3_; Cat. No. 10224350; Fisher Scientific, New Hampshire, USA).

Cell viability/proliferation was determined by adding 10 μl of PrestoBlue® reagent (Cat. No. G8080; Promega, In Vitro Technologies, Madison, WI, USA) to each well for 2 h at 37 °C. Fluorescence was measured on a Synergy 2 Multi-mode microplate reader with an excitation of 535 nm (25 nm bandwidth) and an emission of 615 nm. Cell viability of the test agents was expressed as a percentage of the untreated control at each time point. Data was calculated as a mean ± SD. Paired *t*-tests were used for comparisons and IC_50_ regression analysis conducted in GraphPad PRISM7 (GraphPad Software, San Diego, CA, USA, http://www.graphpad.com).

### Apoptosis assay

Two separate assays measuring caspase 3/7 as a marker of apoptosis were undertaken. In the first the cytotoxic doses of AgNPs (22.5 and 50 μg ml^−1^) were investigated over the short exposure times of 1 and 2 h. The second assay investigated the significantly higher viability of HGF cells when exposed to 0.225 μg ml^−1^ of AgNP and investigate the 4 and 24 h time points. HGFs (*n* = 3) were seeded, as described above, and medium containing AgNPs compared to an equivalent volume of carrier DH_2_O (pH 10.15) as the control. An Apo-one homogeneous caspase 3/7 assay (Cat. No. PMG7790 Promega, In Vitro Technologies, Madison, WI, USA) was used to measure protease activity at an excitation wavelength of 485 nm and an emission of 528 nm on a Synergy 2 Multi-mode microplate reader. Staurosporine (10 μM) was used as a positive control at the recommend time of 6 h.

### RNA harvesting

HGFs (*n* = 3) were seeded at a density of 6000 cellsper cm^2^ in 6-well plates (2 wells per test/control condition) and incubated in 5 ml DMEM/10% FBS at 37 °C for 12 h. The medium was subsequently replaced with 3 ml, per well, of fresh culture medium (DMEM/10% FBS) containing AgNP (0.225 μg ml^−1^) or an equivalent volume of carrier DIH_2_O (pH 10.01) in the controls. The treated HGFs were incubated at 37 °C and cells were harvested with 500 μl of TRIzol (Cat. No. 15596026; Thermo Fisher, MA, USA) per condition at 4, 24, 48 and 72 h and stored at −80 °C until RNA extraction was performed.

### RNA extraction and cDNA

Total RNA was isolated using the Invitrogen Trizol Plus RNA Purification kit and Phasemaker™ Tubes Complete system, using the recommended procedure. Genomic DNA contamination was removed using On-Column PureLink DNase treatment (Ambion, Foster City, CA, USA) and the purity and quantity of RNA was assessed using a NanoVue (GE Healthcare, Little Chalfont, UK). The RNA samples were stored at −80 °C.

Total RNA (300 ng) was used to synthesize cDNA (High Capacity cDNA Reverse Transcription Kit; Gibco Invitrogen). Thermal cycling was in a total volume of 20 μl, as directed by the manufacturer's protocol. The resulting cDNA was diluted to produce 150 μl quantities of 1 ng ml^−1^ using RNase-free H_2_O.

### Gene expression assays were conducted using qRT^2^-PCR technology

Quantitative TaqMan™ real-time PCR (qRT^2^-PCR) single-gene assays were conducted with 10 genes of interest: TP53, GADD45A, CDK1, CCNB1, CDK2, PARP1, AOD2, MAPK8, BCL2, and PCNA. Three housekeeping genes (HKG) of GAPDH, HPRT1, and B2M where screened for normalization. Normfinder (Visual Basic Application applet for Microsoft Excel) was used to determine the optimal normalization gene and determined GAPDH was the most stable with an *M*-value of 0.013. Quantitative RT^2^-PCR efficiencies were calculated by plotting a standard curve for each gene, derived from a five-point dilution series of cDNA, conducted in duplicate. Standard curves had correlation coefficients >0.97 and efficiencies >92% (slope range 2.97 to 3.52). Thermal cycling and detection were performed with a QuantStudio 6 Flex instrument (Applied Biosystems). No cDNA and no reverse transcriptase reactions were included as controls. All the experiments were performed on 3 different HGF cell lines using duplicate wells per sample.

### Statistical analysis

For cell viability and apoptosis, the test and control groups were compared at each time point using paired *t* tests in Graphpad Prism software. Analysis for the gene assays was conducted using the raw quantification cycle (*C*_q_) values of the tested genes normalized against the mean *C*_q_ of the reference gene (GAPDH). The data were analyzed using SABiosciences Microsoft Excel-based PCR Gene Data Analysis template with Graphpad PRISM software. Genes with mean *C*_q_ values of 34 or greater were excluded. Fold regulation in gene expression was determined by comparison of the mean of the normalized expression values between the test and control groups using the ΔΔ*C*_q_ method. Genes with a fold regulation of ±2.0 and a *p* ≤ 0.05 were considered to be significantly regulated.

## Results

### Synthesized AgNPs

**Fig. 1:**
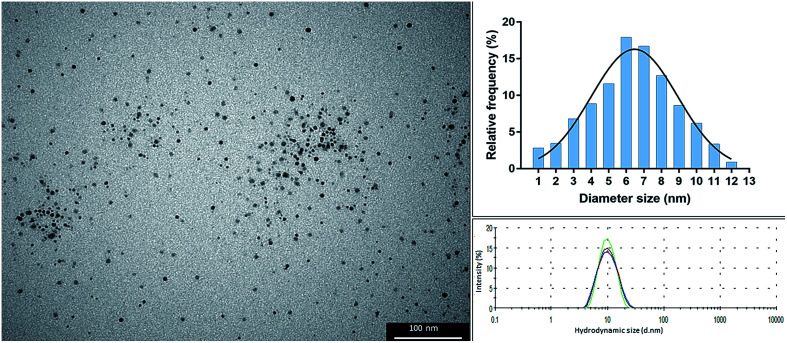
Transmission electron microscopy (TEM) image of alpha lipoic-capped AgNPs (left), histogram of size frequency derived from particle analysis of TEM images (938 particles analysed) (right top), and dynamic light scattering of 4 repeat measurements showing an average hydrodynamic size of 10.7 nm.

The colloidal suspension of alpha lipoic acid-capped AgNPs (solvent H_2_O, pH 10 ± 0.5) was dark yellow-brown in color, which is typical of stable and well dispersed nanoparticles. The particles possessed a hydrodynamic size of 10.7 nm ± 0.050 and polydispersity index of 0.128 ± 0.012. TEM analysis showed that the particles were spherical with an average diameter of 6 nm (range: 1–12 nm; as shown in the size frequency histogram derived from particle analysis of TEM images, Fig. 1).

### Silver nanoparticle aggregation assay

**Fig. 2:**
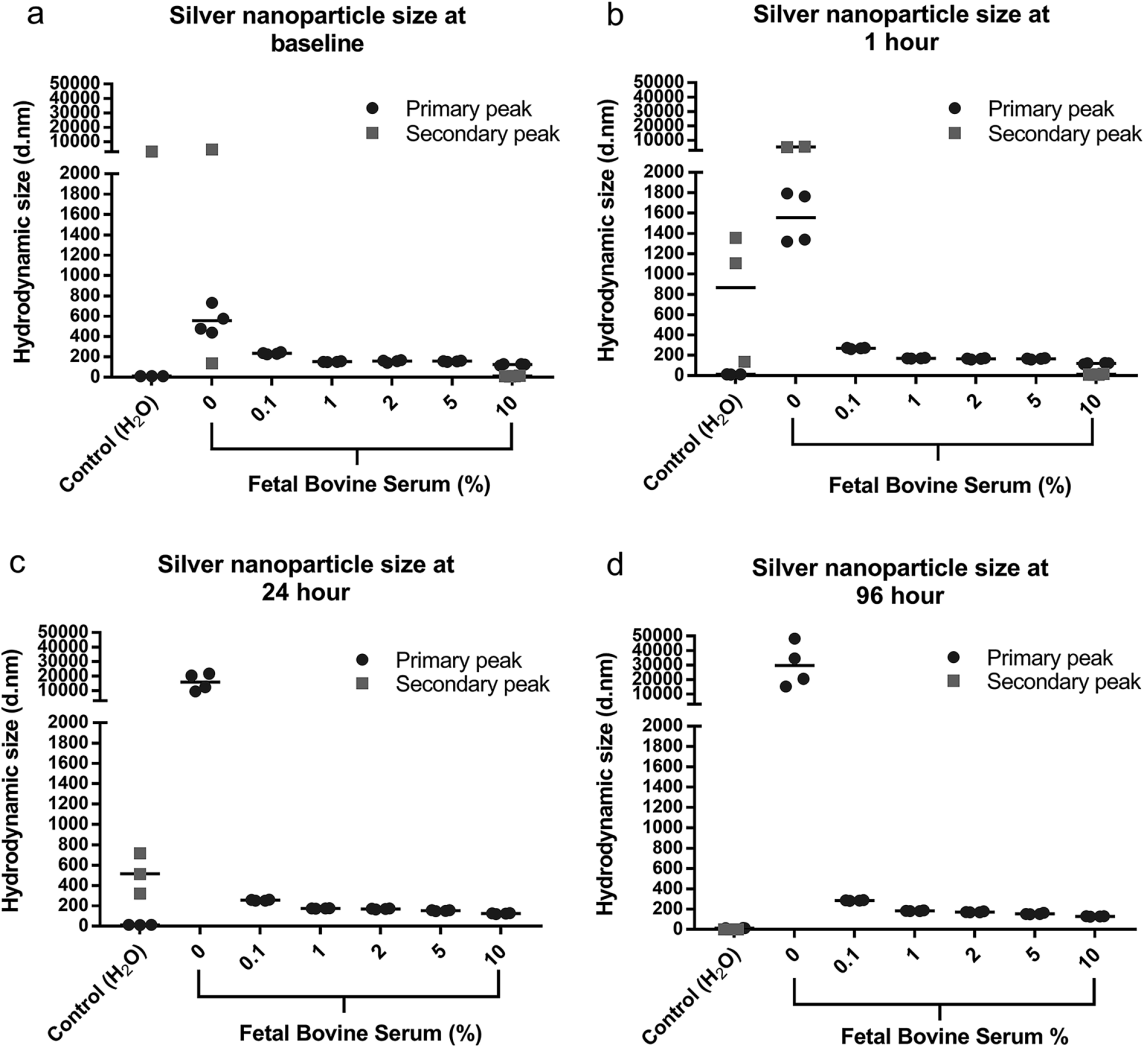
Hydrodynamic particle size (*d* nm) of AgNPs (22.5 μg ml^−1^) dispersed in either DMEM with increasing Fetal Bovine Serum concentration measured at 0, 1, 24, and 96 h using dynamic light scattering.

AgNPs were added to DMEM cell culture media, with increasing amounts of FBS (0–10%) and the particle hydrodynamic size was monitored using DLS. By monitoring the particle size a more accurate aggregation size profile of the AgNPs was determined in different environmental conditions as reflected by the cell culture medium. The size of the original AgNPs as well as this aggregation should be considered when interrupting cytotoxicity and antimicrobial effects. Four measurements were performed per sample, and the particle size with the highest % was considered the primary peak with the particle size with lower % considered the secondary peak. Where only a primary peak is presented, this particle size represented 100% of the nanoparticle suspension, this occurred for all media with ≥0.1% FBS. For suspensions containing <0.1% FBS, two distinct hydrodynamic sizes were detected, which suggested that larger aggregates and varied size distribution was consistent with lower FBS percentages. The distribution of measured sizes and mean of the four repeat samples are presented in Fig. 2.

When AgNPs were dispersed in cell media without FBS, rapid aggregation was observed as a visual precipitation/sedimentation of AgNPs and also as an increase in hydrodynamic diameter size of the AgNPs (Fig. 2 and S1†). With increasing FBS concentration the particles exhibited no visible sedimentation and appeared well dispersed, however the AgNPs continued to demonstrate some increase in hydrodynamic size. A small proportion (6%) of AgNPs maintained their discrete 10 nm size for 1 h when added to DMEM/10% FBS. At 24–96 h AgNPs in 10% FBS possessed a hydrodynamic size of 130 nm, which is representative of the resulting particle size within the cytotoxic and antimicrobial experiments.

### Antimicrobial effects on oral bacteria

The antibacterial properties, as determined by both the MIC and MBC, for AgNPs and AgNO_3_ (as a free-Ag^+^ solution), were evaluated against a range of bacteria *via* a micro-dilution broth assay. All tested bacteria were inhibited and killed within 24 h of incubation with either AgNPs or AgNO_3_ (Table 1, Fig. S2 and S3†). AgNPs and AgNO_3_ had similar inhibition profiles for each bacterium except for *S. aureus* where a higher concentration of AgNPs (12.5 μg ml^−1^ compared to 5 μg ml^−1^ respectively) was necessary. AgNO_3_ displayed greater bactericidal efficacy with MBCs ranging from 2.5–12.5 μg ml^−1^. Consistently higher concentrations of AgNPs were required to achieve bactericidal effects, with MBCs ranging from 5–100 μg ml^−1^. *S. mutans* was the most resistant bacterium of the test species when treated with AgNPs, whereas *E. coli* was the least resistant to both AgNPs and AgNO_3_.

**Table 1:** Minimal inhibitory concentration and minimal bactericidal concentration of thioctic acid-capped AgNPs, and AgNO_3_

Bacterial strain	MIC (μg ml^−1^)		MBC (μg ml^−1^)	
	AgNP	AgNO_3_	AgNP	AgNO_3_
*E. coli* DH5α	2.5	2.5	5	2.5
*S. aureus* NCTC 6571	12.5	5	50	12.5
*P. aeruginosa* OT15	5	5	50	12.5
*S. mutans* UA159	2.5	2.5	100	12.5
*S. mitis* IL8	2.5	2.5	22.5	5
*S. gordonii* DL1	5	5	12.5	12.5

### Cytotoxicity

**Fig. 3:**
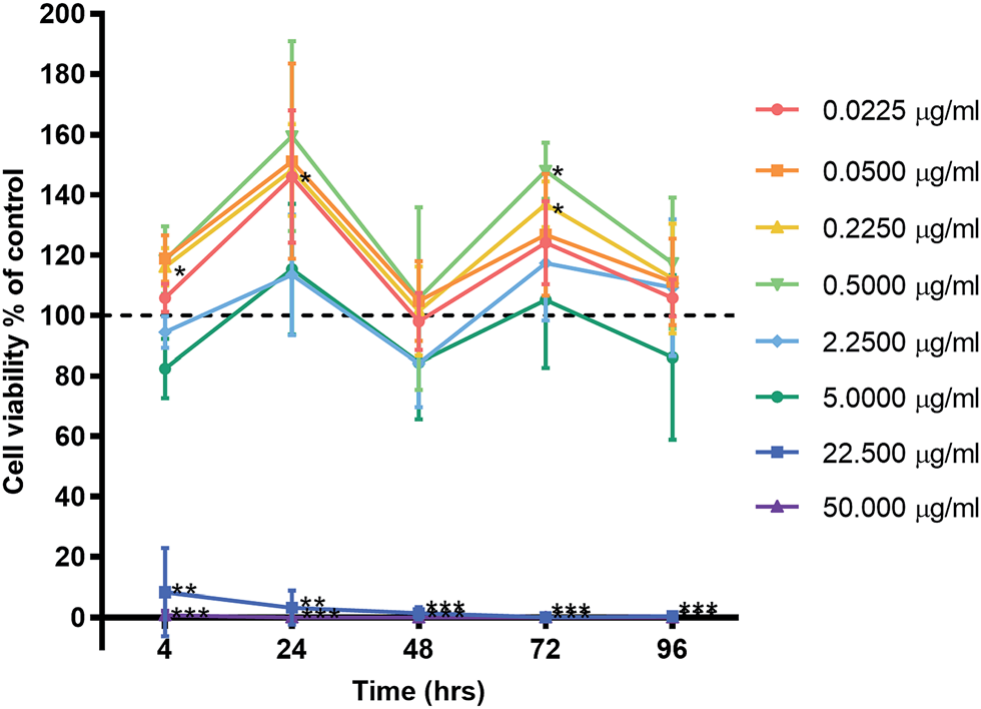
Effect of AgNP concentration on human gingival fibroblast viability. Three HGF cell lines were treated with AgNPs and graphed with the mean ± SD. Dashed line represents the control levels. Significant values *p ≤ 0.05, **p ≤ 0.01, ***p ≤ 0.001.

Primary human gingival fibroblasts (HGF; *n* = 3) were treated with 10-fold dilutions of alpha lipoic acid-capped silver nanoparticles, with 10-dilutions starting at both 50.00 and 22.50 μg ml^−1^ (50.00, 22.50, 5.00, 2.25, 0.50, 0.225, 0.050, and 0.0225 μg ml^−1^). Incubation was at 37 °C for 4, 24, 48, 72, and 96 h. For all time points, ≥22.5 μg ml^−1^ of AgNP resulted in significant cell death (*p* ≤ 0.01) where viable cells ranged from 8.42% ± 14.58 to 0% ± 0.01 (mean ± SD) of controls (Fig. 3). For concentrations ≤ 5 μg ml^−1^ AgNP, a cell cycling effect could be seen with peaks of cell viability at 24 and 72 h. Most notable were concentrations 0.225 and 0.50 μg ml^−1^ of AgNP with the greatest significant increase in cell viability from all tested concentrations. HGF treated with 0.225 μg ml^−1^ AgNP exhibited significantly increased viability at 24 h (148.28% ± 15.27; *p* = 0.0318), 72 h (136.94% ± 7.55; *p* = 0.0137), and 0.5 μg ml^−1^ AgNP at 72 h (148.07% ± 9.38; *p* = 0.0125).

**Fig. 4:**
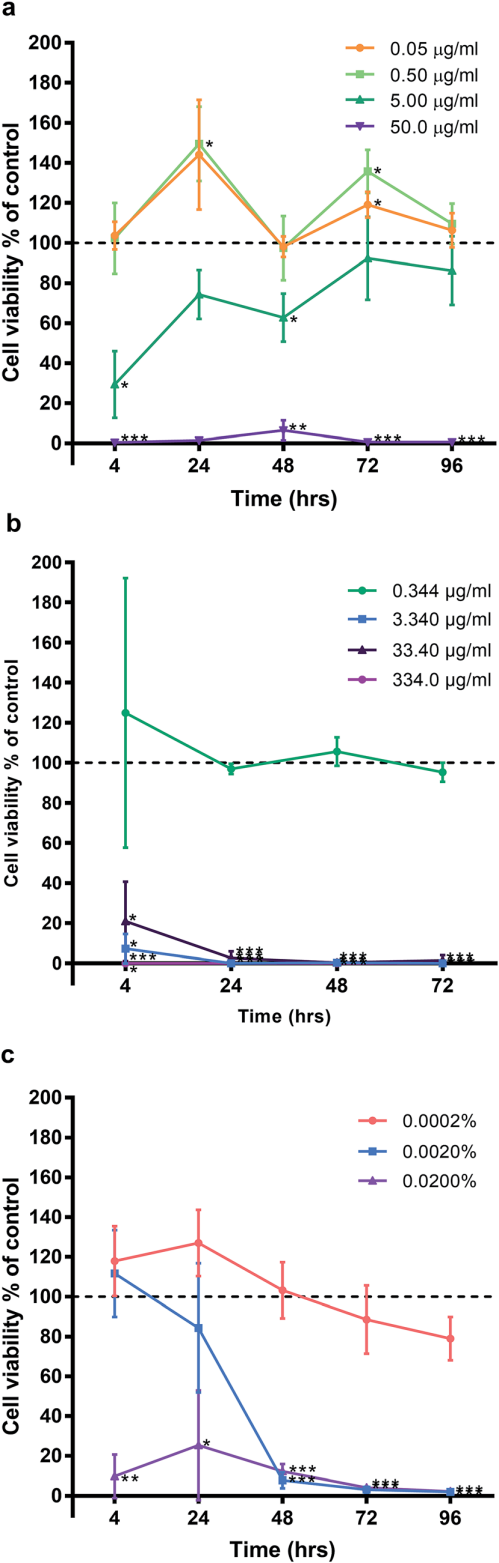
Effect of (a) silver nitrate, (b) silver diamine fluoride, (c) chlorhexidine, on human gingival fibroblast viability. Three HGF cell lines treated with AgNPs and graphed with the mean ± SD. Dashed line represents the control levels. Significant values *p ≤ 0.05, **p ≤ 0.01, ***p ≤ 0.001.

HGF treated with AgNO_3_, a precursor to silver compounds, were also examined at 10-fold dilutions starting from 50.00 μg ml^−1^ (50.00, 5.00, 0.50, 0.05 μg ml^−1^) for the same time points. Similar patterns of cell cycling was observed (Fig. 4a). All time points, 50.0 μg ml^−1^ AgNO_3_ significantly decreased HGF cell viability (0.43% ± 0.75 to 39.70% ± 66.44) as compared to controls at 100%. While 5.00 μg ml^−1^ AgNO_3_ at 4 h began with a significant decrease in cell viability (29.34% ± 16.69; *p* = 0.0181), over time cells exhibited a statistical improvement in viability (48 h: 68.80% ± 11.99; *p* = 0.0329), to levels closer to control. At lower concentrations a significant increase in cell viability was detected and this was most marked for 0.50 μg ml^−1^ AgNO_3_ at 24 (149.53% ± 18.67, *p* = 0.0442) and 72 h (135.83% ± 10.85 *p* = 0.0292).

Experiments with SDF and CHX as clinical antimicrobials, also saw decreased HGF cell viability when examined under the same experimental conditions (Fig. 4b and c respectively). SDF was tested between concentrations 0.344–334.0 μg ml^−1^ and CHX 0.0002–0.02% in 10-fold dilutions for the same time points of 4, 24, 48, and 72 h. Concentrations ≥ 3.340 μg ml^−1^ SDF on HGF resulted in a significant decrease in cell viability at 4 h (7.26% ± 2.60, *p* = 0.0021), and at 96 h, there was statistically significant cell death (0.07% ± 0.01, *p* = 0.0000002; Fig. 3). Of the tested doses only 0.344 μg ml^−1^ of SDF resulted in sustained cell viability when compared to control wells (124.99% ± 67.23, *p* = 0.59, at 4 h; 95.23% ± 0.01. *p* = 0.23, at 72 h). Like SDF, treatment of HGF with CHX resulted in a significant reduction in cell viability (Fig. 4c). A dose of 0.02% CHX significantly reduced cell viability at 4 h (9.80% ± 10.96; *p* = 0.0049) when compared to control (100%). While 0.002% CHX was initially similar to control at 4 h (111.76% ± 21.86, *p* = 0.45), after 48 h of treatment there was a significant decrease of cell viability (7.83% ± 4.05, *p* = 0.0006). Over time all doses with CHX were found to result in a decrease in cell viability. The dose response curves tested related to the known clinical efficacy of the antimicrobial agents, which for SDF was analyzed by ICP-MS and found to be 334 000 μg ml^−1^. CHX is used as a 0.2% mouthwash, while the AgNO_3_ dose curve reflected the range used for testing AgNP. Sustained exposure for 96 h (72 h for SDF only) resulted in significant decreases in HGF cell viability with threshold doses of: 5 μg ml^−1^ AgNO_3_ (86.22% ± 17.17, *p* = 0.3), 3.4 μg ml^−1^ SDF (95.23% ± 4.84, *p* = 0.23), and 0.0002% CHX (79.03% ± 10.94, *p* = 0.079). There was a similar pattern of a concentration dependent cell cycling effect for AgNP, AgNO_3_, SDF but not for CHX. There was no significant effect of water (pH 10.01), as the Ag carrier on HGF, apart from at 96 h when high amounts of carrier resulted in a very slight but significant increase in cell viability when compared to control (106.95% ± 2.05, *p* = 0.0277; Fig. 9).

### Cell morphology

HGF cell morphology appeared to alter from the control cell morphology with increasing AgNP dosage as shown in Fig. S4 and S5.† Control HGF cells were elongated with a centrally placed nucleus and evidence of cell division was observed. There were noticeable similarities of cell morphology and percentage confluence of the cells between 0.0225 and 5.00 μg ml^−1^ when compared to the control treatment group. With increasing concentrations of AgNP less cells were evident and of those present a higher number were rounded, or had irregular shapes, and cell debris was observed. At AgNP concentrations of 22.5 and 50 μg ml^−1^, the HGF cell membranes had lost definition and linearity, which correlated to a lack of cell function. AgNP aggregates and precipitation were noticeable in treated samples >5 μg ml^−1^.

### IC_50_ and apoptosis

**Fig. 5:**
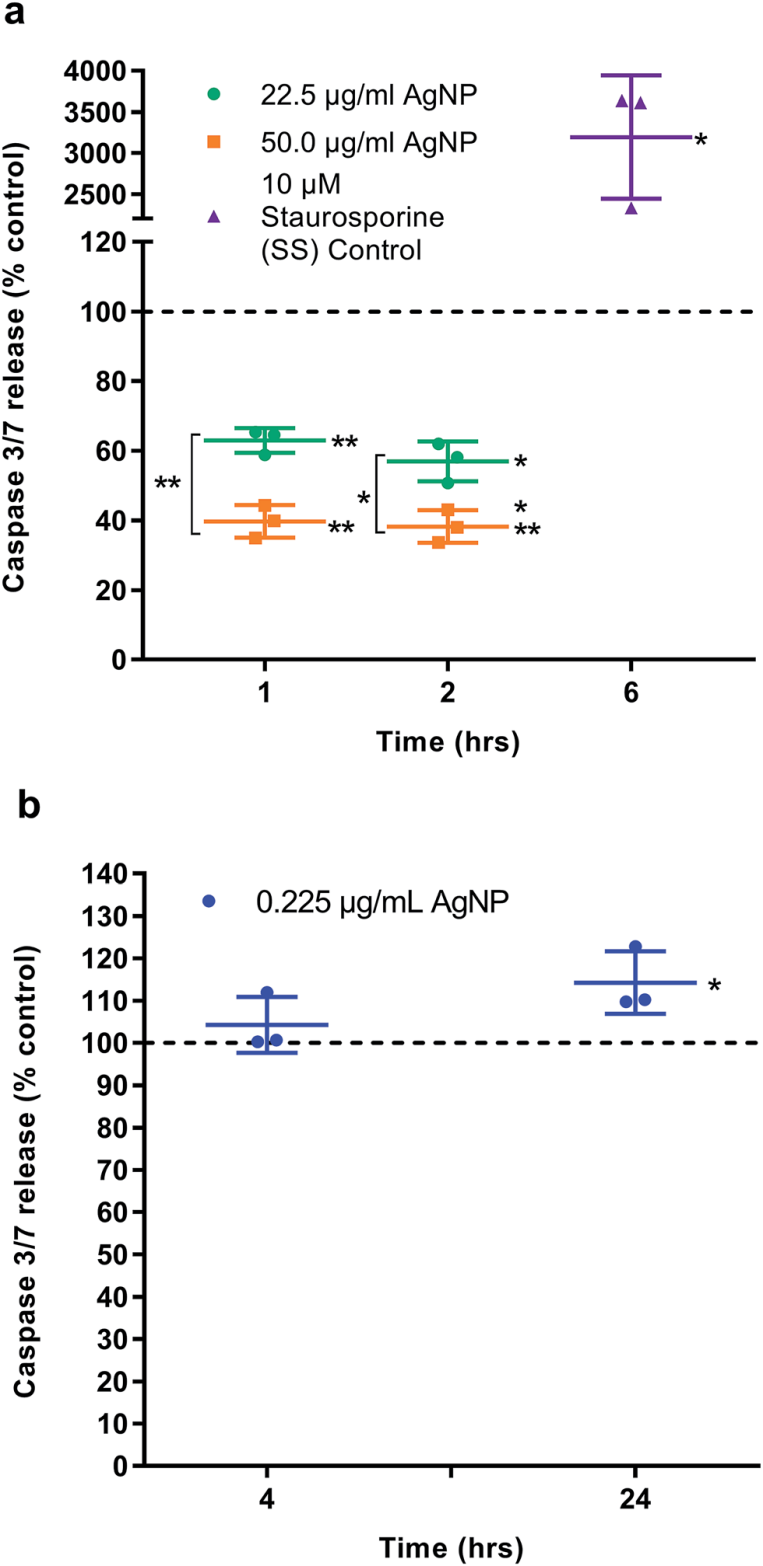
Caspase 3/7 activity. Apoptosis was examined in HGFs (*n* = 3) treated with AgNPs (a) 22.5 and 50 μg ml^−1^ at 1 and 2 h, (b) 0.225 μg ml^−1^ at 4 and 24 h. Staurosporine (SS) was included as a positive control. Dashed line represents the control levels. Results expressed as mean ± SD. Significant values *p ≤ 0.05, **p ≤ 0.01.

The half maximal inhibitory concentration (IC_50_) of AgNPs on HGFs was determined as 10.2 μg ml^−1^ at 4 h and 10.6 μg ml^−1^ at 96 h (Fig. S6†). The caspase 3/7 assay was validated using SS as a positive control (Fig. 5a). AgNPs appears to result in a statistically significant anti-apoptotic effect, as measured by caspase 3/7 reduction, at 22.5 μg ml^−1^ (1 h: 62.9% ± 3.56, *p* = 0.0031; 2 h: 56.9% ± 5.47, *p* = 0.0059) and 50 μg ml^−1^ (1 h: 39.8% ± 4.67, *p* = 0.002; 2 h: 38.3% ± 4.72, *p* = 0.002) as compared to control. There was also a significant dose response when comparing 22.5 to 50.0 μg ml^−1^ of AgNP (1 h: *p* = 0.002, 2 h: 0.0032; Fig. 8). However, when correlating the caspase 3/7 production to the significantly lower cell viability when treating HGFs with 22.5 and 50 μg ml^−1^ of AgNPs, it was considered that the reduced caspase 3/7 production was due to the lower number of viable cells. Further investigation of caspase 3/7 production using a non-cytotoxic AgNP concentration, 0.225 μg ml^−1^, demonstrated no significant effect of AgNPs on caspase 3/7 production when compared to the controls (4 h: 104% ± 6.64, *p* = 0.38; Fig. 5b). The slightly increased caspase 3/7 production at 24 h corresponded to a significantly higher proliferation of cells at this time point (24 h: 106.8% ± 2.63, *p* = 0.046). AgNPs therefore did not appear to affect the apoptosis pathway (neither apoptotic inducing or anti-apoptotic).

### Relative qRT^2^-PCR determination of cell cycle and oxidative genes

**Fig. 6:**
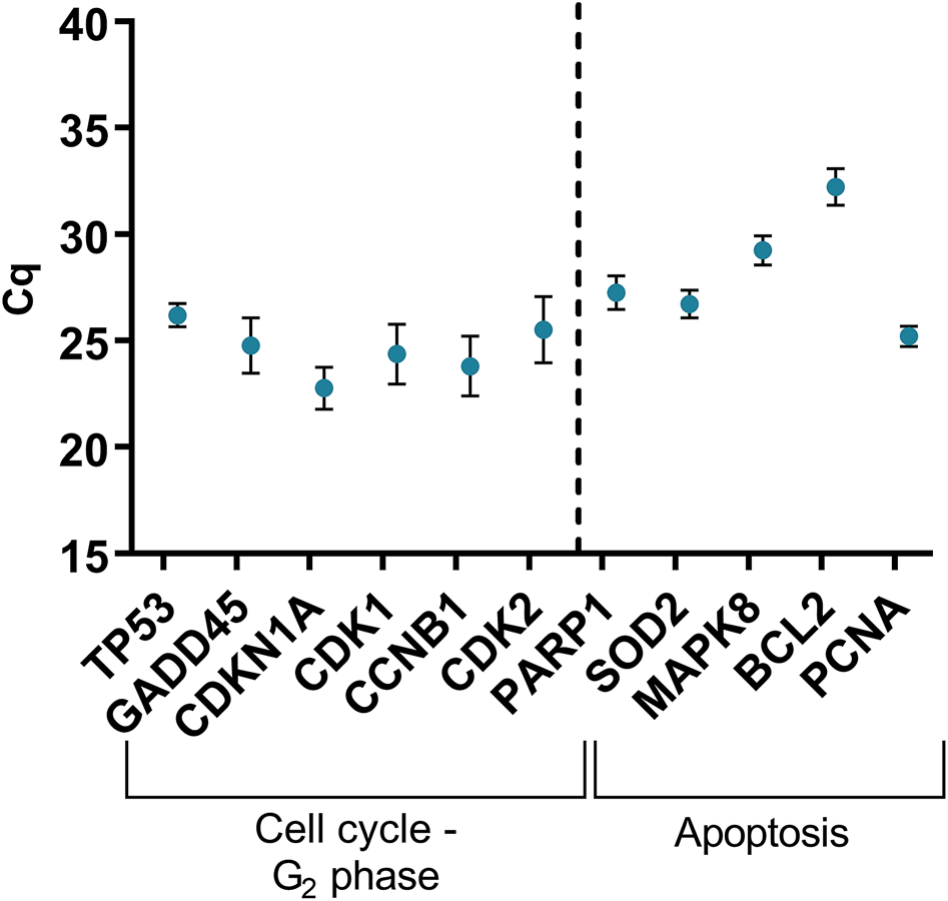
*C*_q_ values of genes of interest expressed in HGFs (*n* = 3) treated with 0.225 μg ml^−1^ AgNPs for 4 h.

**Fig. 7:**
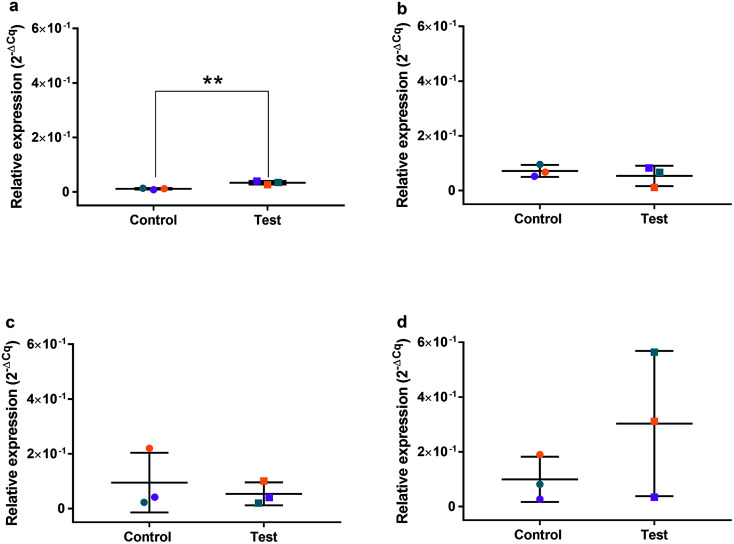
Relative qRT^2^-PCR expression of TP53 with 0.225 μg ml^−1^ AgNPs or control conditions (DH_2_O pH 10.01). RNA was isolated from HGFs (*n* = 3) at (a) 4, (b) 24, (c) 48 and (d) 72 h. Results expressed as mean ± SD. *p-value ≤ 0.05, **p ≤ 0.01.

**Fig. 8:**
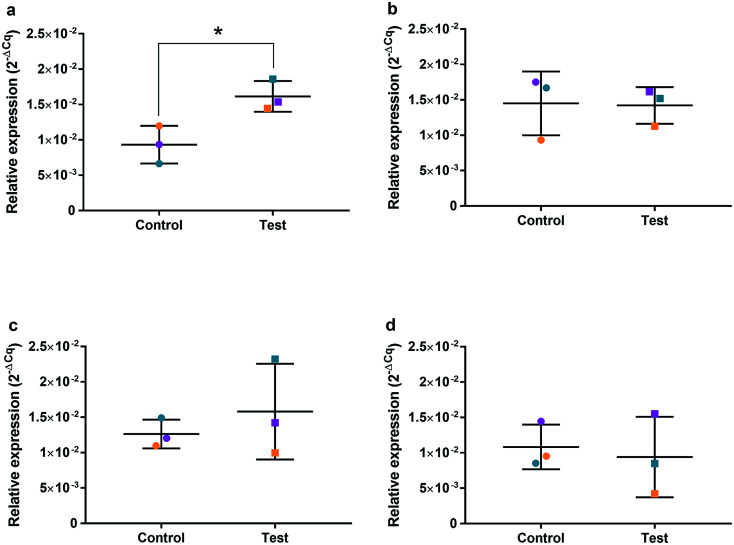
Relative qRT^2^-PCR expression of PARP1 with 0.225 μg ml^−1^ AgNPs or control conditions (DH_2_O pH 10.01). RNA was isolated from HGFs (*n* = 3) at (a) 4, (b) 24, (c) 48 and (d) 72 h. Results expressed as mean ± SD. *p-value ≤ 0.05, **p ≤ 0.01.

**Fig. 9:**
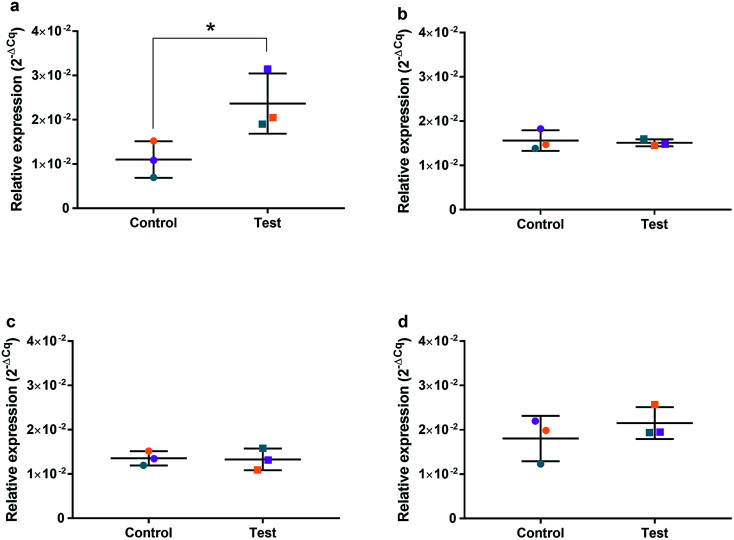
Relative qRT^2^-PCR expression of SOD2 with 0.225 μg ml^−1^ AgNPs or control conditions (DH_2_O pH 10.01). RNA was isolated from HGFs (*n* = 3) at (a) 4, (b) 24, (c) 48 and (d) 72 h. Results expressed as mean ± SD. *p-value ≤ 0.05, **p ≤ 0.01.

**Fig. 10:**
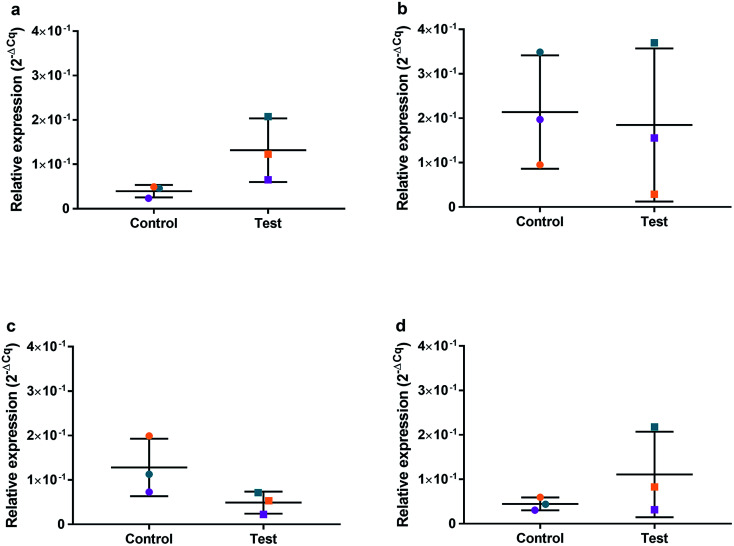
Relative qRT^2^-PCR expression of CDK1 with 0.225 μg ml^−1^ AgNPs or control conditions (DH_2_O pH 10.01). RNA was isolated from HGFs (*n* = 3) at (a) 4, (b) 24, (c) 48 and (d) 72 h. Results expressed as mean ± SD. *p-value ≤ 0.05, **p ≤ 0.01.

HGFs were treated with a non-cytotoxic concentration of AgNPs at 0.225 μg ml^−1^ and which was shown to induce a statistically significant cell cycling effect. RNA was collected at 4, 24, 48, and 96 h and was analyzed using qRT^2^-PCR to determine relative fold changes of gene expression of 11 genes and one housekeeping gene compared to non-treated HGF controls. *C*_q_ gene expression levels of TP53, GADD45, CDKN1A, CDK1, CCNB1, CDK2, PARP1, SOD2, MAPK8, BCL2, PCNA, and housekeeping gene GAPDH were studied at 4, 24, 48, and 96 h times points and overall showed lower expression levels of apoptotic related genes and a consistently higher expression of growth cycle related genes (Fig. 6). Significantly upregulate genes at 4 h included TP53 (*p* = 0.0058), PARP1 (*p* = 0.026) and SOD2 (*p* = 0.051), while CDK1 (*p* = 0.094) also showed some upregulation (Fig. 7a–10a). The upregulation of these genes was observed at 4 h only and values returned control levels at 24 h onward (Fig. 7–10). The results suggest that when HGFs are treated with AgNPs this induces an initial oxidative stress; however, the cells were able to recover when exposed for longer time periods.

## Discussion

The present study has shown that alpha lipoic acid-capped AgNPs possess inhibitory antimicrobial effects at low concentrations (2.5–12.5 μg ml^−1^). Concentration dependent cytotoxic effects were observed in HGFs treated with alpha lipoic acid-capped AgNP; no adverse cell viability changes were observed at lower concentrations ≤ 5 μg ml^−1^ whereas at higher concentrations ≥ 12.5 μg ml^−1^ a significant reduction in cell viability was seen. Over 4 days, no cumulative cytotoxic effect of AgNP treatment on HGFs was observed. AgNP treatment of HGFs demonstrated a less cytotoxic profile compared to clinical controls: silver diamine fluoride (cytotoxic concentration: 3.34 μg ml^−1^; clinical application concentration: 334 000 μg ml^−1^) and chlorhexidine (cytotoxic concentration: 0.02%; clinical application concentration: 0.2%). A similar cytotoxicity profile of AgNPs compared to AgNO_3_ was found, suggesting that the toxic effects of AgNPs may be associated with free released Ag^+^ ions.

The effects of AgNPs at both high (22.5 and 50 μg ml^−1^) and low (0.225 μg ml^−1^) dose on caspase 3/7 production found no induction, suggesting the mechanism of cell death was not apoptosis. A cell cycling response over time was seen with both AgNPs and AgNO_3_, even at very low doses. Furthermore, it was determined that AgNP treatment of HGFs upregulated TP53, PARP1, SOD2, and CDK1 gene expression at 4 h only, and not at the later timepoints examined, suggesting an initial oxidative shock of cells with a subsequent recovery ≥ 24 h.

### Cytotoxicity

*In vitro* cytotoxicity testing on HGF cells is a well-established method and HGFs are the most abundant structural cell in periodontal tissue necessary for oral wound healing.^27,28^ Novel antimicrobial agents for use in periodontal treatment will come into direct contact with oral tissues and therefore require *in vitro* assessment on HGF cells. This study used primary HGFs to examine the possible cytotoxic effects of alpha lipoic acid-capped AgNPs as a potential antimicrobial agent and compared it to other sources of Ag and chlorhexidine.

The cytotoxic effects of AgNPs have been demonstrated on HGF cells previously in an attempt to elucidate their toxicity toward periodontal tissues. Overall it is generally accepted that a smaller sized particle (≤10 nm) are more cytotoxic to both bacteria and human cells;^3^ within this study, the AgNPs were 6 nm (1–12 nm) in diameter. Low concentrations of AgNPs (≤16 μg ml^−1^) have been shown in literature to have little influence on the proliferation of cells, whereas higher concentrations (≥34 μg ml^−1^) can alter cell morphology, inhibit proliferation, led to cell cycle arrest at G1/S and induced apoptosis.^19^ This investigation determined an AgNP IC_50_ of 10.2 μg ml^−1^ at 4 h, and 10.6 μg ml^−1^ at 96 h, which showed a lack of cumulative toxic effect of AgNP on HGF cells; and also suggested that the initial damage is within the first 4 h of AgNP application. Proliferation of cells and appearance of cell morphology was consistent with the control cells at ≤5 μg ml^−1^, whereas both features were affected at concentrations ≥ 12.5 μg ml^−1^. It has been proposed that AgNPs induce oxidative stress on HGF cells, *via* ROS generation and lipid peroxidation in a concentration dependent manner.^29,30^ ROS produce an impaired physiological function due to cellular damage of DNA proteins, phospholipids, and other macromolecules.^31^ ROS damage may explain why the HGF cell membranes appeared undefined and nonlinear when observed, potentially caused by lipid peroxidation to the membrane structures leading to a lack of cellular integrity and loss of cell viability.

It was apparent through our studies that the alpha lipoic acid capped AgNPs, when compared to other clinically used controls, demonstrated a significantly reduced toxicity relative to their dose for clinical efficacy. Chlorhexidine at 0.02%, produced a significant reduction in HGF cell viability when treated over a 4 hour period. Chlorhexidine is considered the gold standard antiseptic used in dentistry and is known to be an effective antimicrobial agent for reduction of both plaque and gingivitis. It is typically used clinically at 0.2% for mouthwashes and irrigation treatment and demonstrates substantivity within the oral cavity (8–12 weeks in hard dental tissues,^27,32^ 12–24 h for soft oral tissues^33^). Silver diamine fluoride demonstrated cytotoxicity at 3.34 μg ml^−1^ with a 4 hour treatment on HGF cells. The commercially available silver diamine fluoride product was analyzed by ICP-MS for silver concentration and was found to be 334 000 μg ml^−1^, 100 000 times more than the cytotoxic concentration observed for HGF cells. Although SDF is formulated for dental caries only, particularly in children, our study shows a high level of cellular cytotoxicity toward HGF cells and therefore gingival contact should be minimized when using SDF; although no adverse effects, bar mild reddening of the gingiva for 24 hours^29^ have been reported in clinical studies.^34^

There has been a resurgence in the use of silver nitrate in combination with sodium as a non-invasive treatment in children for arresting caries. In this study, silver nitrate was used as a silver comparative of free Ag^+^ ions at the same concentration of silver delivered *via* AgNPs. AgNO_3_ was slightly more cytotoxic toward HGF cells when compared to AgNPs, interestingly, a similar time-dependent modulation of cell proliferation was seen at non-cytotoxic concentrations ≤ 5 μg ml^−1^ for silver nitrate as seen with AgNPs. At 5 μg ml^−1^, silver nitrate showed an initial reduction of cell viability to 30% of the control, HGF cells subsequently recovered over 96 hours to 80% viability.

### Antimicrobial properties

Both AgNP and AgNO_3_ were active against a variety of organisms including oral-associated bacteria, however AgNO_3_ demonstrated a slightly higher bactericidal action. The similarity of antimicrobial effects suggests a shared antimicrobial mechanism of action of AgNP and AgNO_3_, which is most likely due to the ionic Ag^+^ release attributing to the observed toxicity. The lower bactericidal effect of AgNPs may be due to an alter release profile of Ag^+^ with potential a slower but longer profile due to an increase in size of AgNP assemblies. There is also the possibility that the AgNPs are ‘disguised’ by the surface adsorption of protein leading to a reduction in ionic Ag^+^ release and therefore subsequent toxic effect. A similar study using 10 nm alpha lipoic acid-capped AgNPs reports slightly higher MIC values, but utilized different bacterial species which might be more prolific, with values ranging from 5–40 μg ml^−1^ which they report as non-cytotoxic concentrations toward HGF cells.^17^ The authors also reported the lack of efficiency of AgNPs in preventing biofilm growth, requiring 40 μg ml^−1^ AgNP for *S. mutans* ATCC 29175 whereas in this study *S. mutans* had a significantly higher MBC of 100 μg ml^−1^; the MBC difference may be due to the processes involved in nanoparticle synthesis having significant impact on the resulting antimicrobial effects, and the differences between the bacterial assays, such as media. Interestingly it was reported that bacteria were more susceptible to AgNPs rather than to the reference solutions of AgNO_3_ ^18^ however our studies suggest that AgNO_3_ is more antimicrobial which is in line with the conclusion that the observed toxic effects are due to the concentration of bioavailable Ag^+^ ions.^28^ In particular we note that alpha lipoic acid-capped AgNPs possess higher MIC/MBC values than some AgNPs seen in literature, however are considered less cytotoxic due to alpha lipoic acid exhibiting radical scavenging activity and thus antioxidant properties.^34^

### Silver nanoparticle aggregation in cell media

The hydrodynamic size of the AgNPs was monitored over time in cell culture medium DMEM with increasing FBS concentration, using DLS. Hydrodynamic size is a measurement of the nanoparticle and the closely associated solvent molecules within a particular suspension, as such the hydrodynamic size is usually larger than the core nanoparticle size.^35^ It was evident that the alpha lipoic acid-capped AgNPs became unstable through molecule/protein adsorption and/or loss of surface functionality, and therefore repulsion when introduced to the media. The loss of nanoparticle stability resulted in the formation of aggregates and a larger reported hydrodynamic size. The AgNPs appeared more unstable in DMEM without FBS as observed by larger aggregate sizes. Additionally, these larger aggregates were not colloidally dispersed and rapidly sedimented. It is likely that this increase in size was due to AgNPs interacting with cations within the media, permitting cross linking between the available carboxylate group on the exterior of the particle, an intermediate cation, and an adjacent terminal carboxylate on an adjacent particle. The increasing concentration of FBS appeared to limit the size of the AgNP assemblies and aided particle dispersity. In particular, 10% FBS maintained a small percentage of the discrete AgNP size (∼10 nm) over 1 h with the remaining particles forming small assemblies. It was concluded that although antimicrobial effects and cytotoxic effects are typically reported for the as-synthesized AgNPs, the effects are actually highly specific to the media used for assays^3^ and in this study the presence of serum proteins was most ideal for maintaining the original AgNP size.

### Caspase 3/7 and apoptosis

The activation and expression of caspase-3 and -7 plays a central role in cell apoptosis, and therefore the determination of a significant change in regulation can be indicative of either anti-apoptotic or apoptotic inducing environments/materials.^35–37^ Apoptotic processes are usually viewed as a natural, cell-programmed death that occurs as part of normal cell turnover in healthy adult tissues.^4^ Necrotic death results in a release of inflammatory cellular contents, which can lead to decreased blood flow and tissue damage. Although AgNPs at cytotoxic concentrations (22.5 μg ml^−1^ and 50 μg ml^−1^) appeared to significantly reduce caspase 3/7 regulation, this observation was correlated to a significant reduction in cell viability of HGFs. A non-cytotoxic AgNP concentration, 0.255 μg ml^−1^, that demonstrated significant cell modulation from the control, was used to treat HGF cells and investigate caspase 3/7 production; it was found that there was no significant variation of caspase 3/7 production from the control HGFs. The findings suggest that when high dose AgNPs are cytotoxic to HGF cells the mechanism of death is not *via* apoptosis.

### Quantitative RT^2^-PCR

To investigate the effects of AgNPs on HGF, pathway specific genes were selected for G1/S and G2/M cell cycle control, apoptosis, and ROS production. It is reported that cellular apoptosis has occurred in both bacterial cells and human cells that have been treated with AgNPs and that ROS production from the surface of the AgNPs is a contributing aspect of the observed toxicity.^36–38^ Within this study, HGF treatment with 0.225 μg ml^−1^ AgNPs produced an upregulation of TP53 (*p* = 0.0058), PARP1 (*p* = 0.026), SOD2 (*p* = 0.051), and CDK1 (*p* = 0.093) genes at 4 h only. At subsequent time points, 24, 72 and 96 h, all studied genes were unaffected by AgNP treatment, with no significant change from control levels. The time-dependent change in expression observed here was consistent with cell synchronisation, in which the cells undergo an initial environmental stress to control a uniform growth phase throughout the cell population. This would suggest that the AgNPs are acting as an initial transient stress inducer which the HGF cells are able to recover from and may be a reason why we observe modulation of cell proliferation when compared to control cells.

The upregulation of the particular genes reported suggests ROS mediated stresses on cells with an effect on the G2/M growth phase. When ROS production or exogenous ROS levels increases above normal cellular levels, antioxidant genes are upregulated in order to protect the cell. Superoxide dismutase 2 (SOD2) is one of these antioxidant genes and acts to convert superoxide to hydrogen peroxide which can be further metabolized by catalases.^37,38^ SOD2 also affects TP53 regulation in stimulating apoptotic events.^17^ In addition to SOD2 indicating cell oxidative stress, PARP1 mediates apoptosis and is commonly activated in response to oxidative stress.^39^ In particular, PARP1 activation is an immediate response to metabolic, chemical or radiation-induced DNA single strand break damage; PARP1 then utilizes ATP to repair the damaged DNA and subsequent depletion of ATP can cause cellular necrosis.^19^ PARP1 has commonly been found to be upregulated in AgNP treated cell lines, and is a marker for an oxidative stress response.^33^ TP53 is a core molecule mediating G2/M and G1/S checkpoint activation in response to DNA damage.^21^ G2/M phase arrest acts as a protective barrier for cell DNA damage repair before cells enter mitosis. Reinforcing the resulting G2/M effects of AgNPs on HGFs is the upregulation of CDK1. CDK1 plays key roles in the cell cycle G2/M phase regulation network and is critical for cell-cycle progression through G2 to M phase. Supporting our finding are reports of Ag-mediated DNA damage resulting in G2/M cell cycle arrest.^21^ The lack of regulation change of GADD45, CDK2M and CDK2 when HGF cells were treated with AgNPs compared to the control, suggests that AgNPs may not affect the functioning of the G1-S phase of the cell cycle. It is therefore likely that AgNPs cause an ROS imbalance in cells, which at non-cytotoxic concentrations can be mitigated by the upregulation of ROS antioxidant genes, such as SOD2, and DNA repair genes and was further reinforced in the synchronisation of cells seen in cell viability experiments.

## Conclusion

It was concluded that AgNPs can be used at an appropriate concentration to inhibit bacteria, without major cytotoxic effects toward human gingival fibroblasts and therefore they may serve as a less toxic antimicrobial for incorporated into biomaterials, particularly when compared to chlorhexidine and silver diamine fluoride.

## Conflicts of interest

G. C. Cotton holds shares in Silventum Ltd and is an inventor on patent applications relating to silver nanoparticle technology. W. J. Duncan is also an inventor on a patent application relating to silver nanoparticle technology.

## Supplementary Material

RA-009-C9RA00613C-s001
