# Supplementary material for: Efficacy and safety of alpha lipoic acid-capped silver nanoparticles for oral applications
Source: RSC Adv. 2019 Feb 28;9(12):6973–85. doi: 10.1039/c9ra00613c (PMC9061105; doi:10.1039/c9ra00613c)
Supplement: RA-009-C9RA00613C-s001 [file RA-009-C9RA00613C-s001.pdf]

Electronic Supplementary Material (ESI) for RSC Advances.
This journal is © The Royal Society of Chemistry 2019

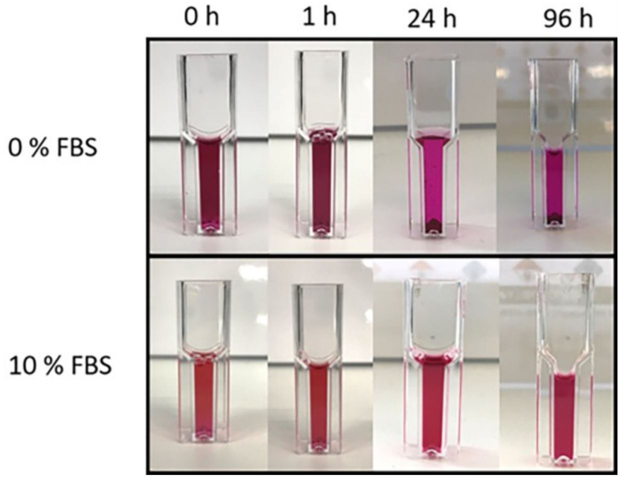

Supplementary Figure S1. Visual sedimentation of AgNPs dispersed in DMEM/0% FBS over 96 h and FBS mediated stabilization of AgNPs in DMEM/10% FBS.

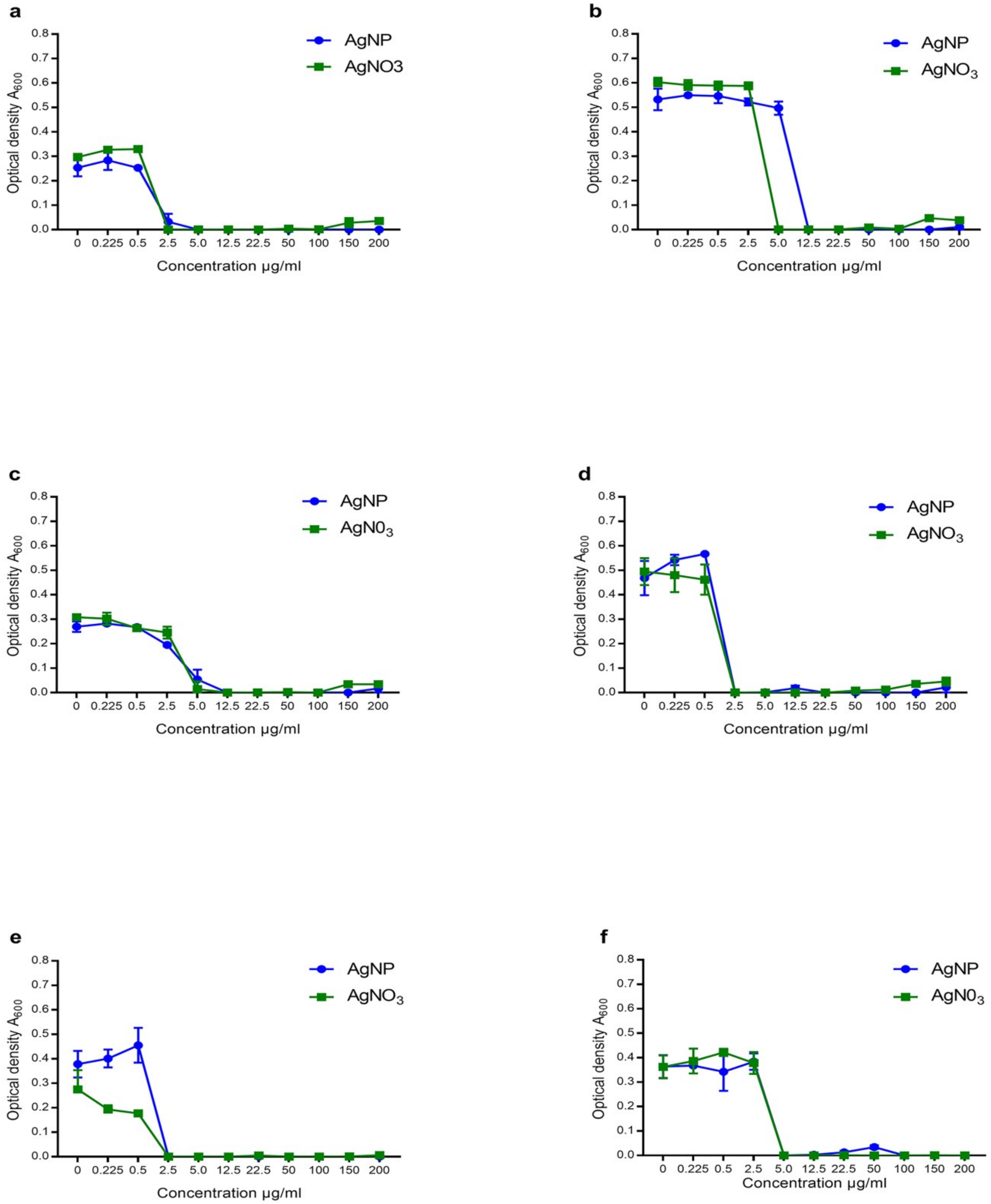

Supplementary Figure S2. Minimal inhibitory concentration, determined by optical density (Abs₆₀₀), of increasing AgNP concentration on (a) E.<i>coli</i> (b) S.<i>aureus</i> (c) P.<i>aeruginosa</i> (d) S.<i>mutans</i> (e) S.<i>mitis</i> (f) S.<i>gordonii</i>.

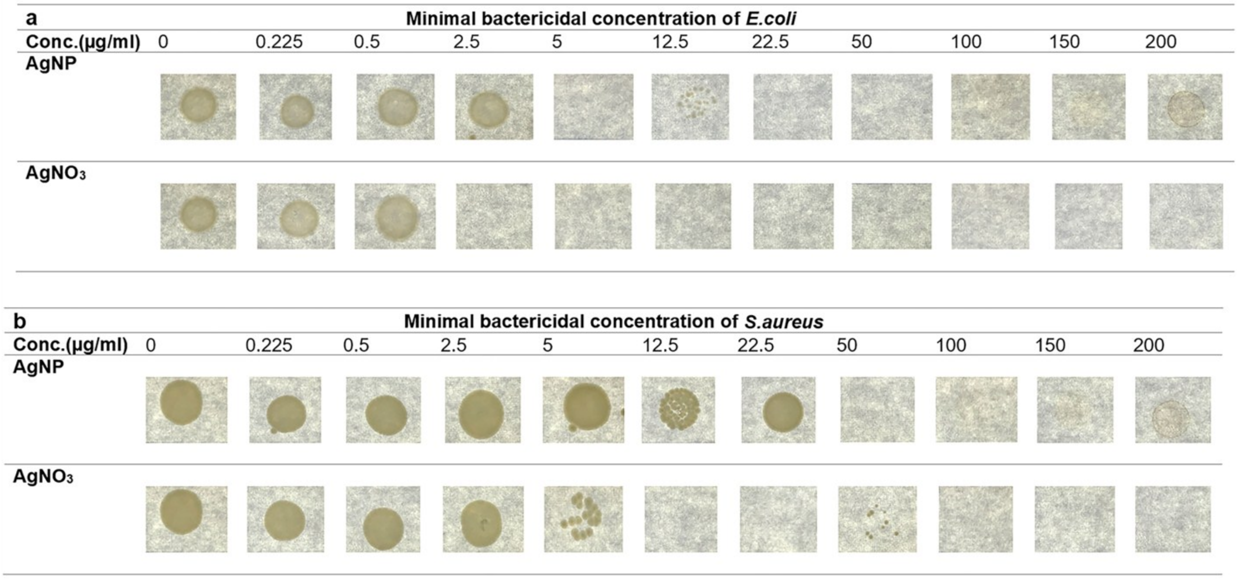

Supplementary Figure S3. Minimal bactericidal concentration, determined by re-plating bacterial cultures subjected to increasing AgNP concentration post 24 h incubation (a) E<i>.coli </i>(b) S.<i>aureus </i>

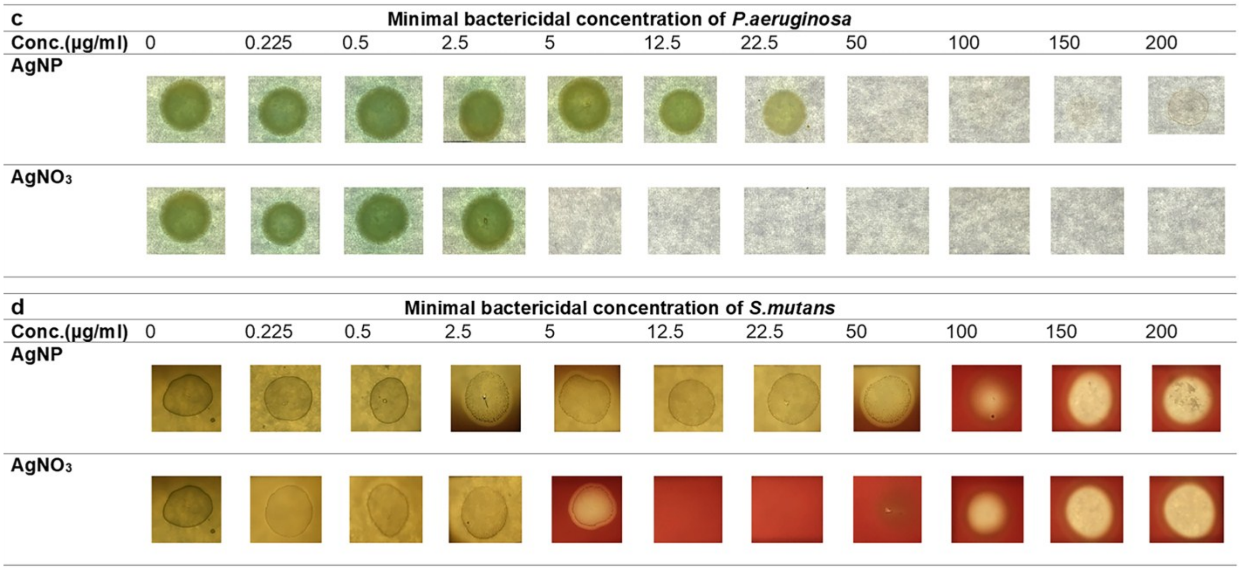

Supplementary Figure S3. Minimal bactericidal concentration, determined by re-plating bacterial cultures subjected to increasing AgNP concentration post 24 h incubation (c) P.<i>aeruginosa</i> (d) S.<i>mutans</i>.

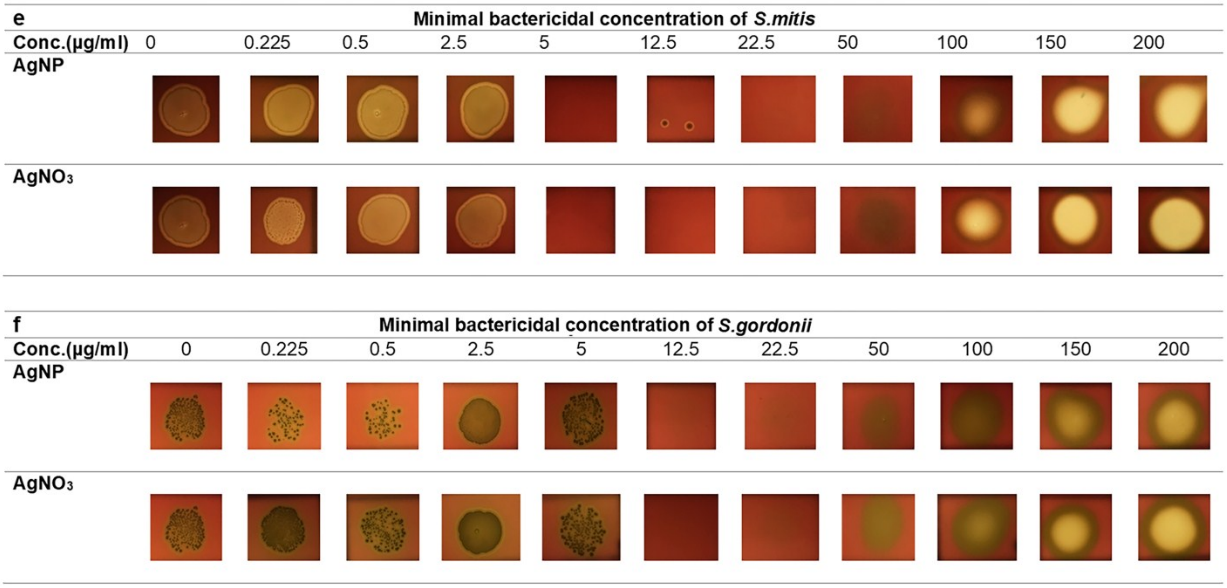

Supplementary Figure S3. Minimal bactericidal concentration, determined by re-plating bacterial cultures subjected to increasing AgNP concentration post 24 h incubation (e) S.<i>mitis</i> (f) S.<i>gordonii</i>.

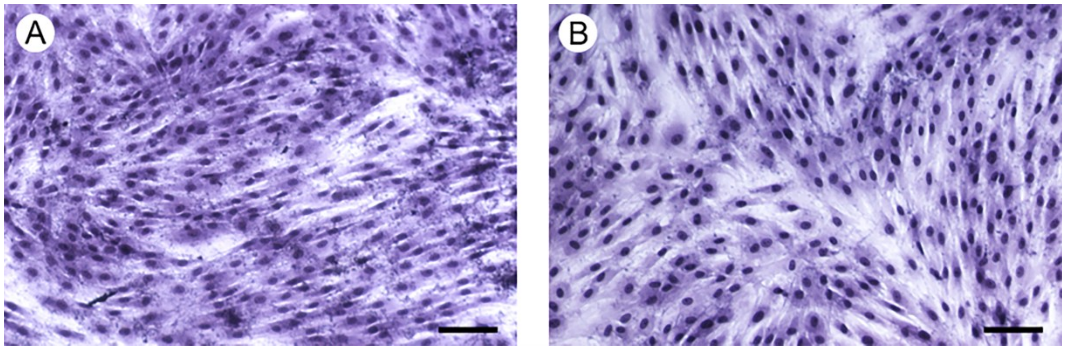

Supplementary Figure S4. Light microscopy images of the cellular morphology of control HGF cells at 96 h (a) cells in DMEM/10% FBS media (b) cells with 22.5 µg/mL alkaline DI-H₂O (pH10.01). Scale bar = 100 µm.

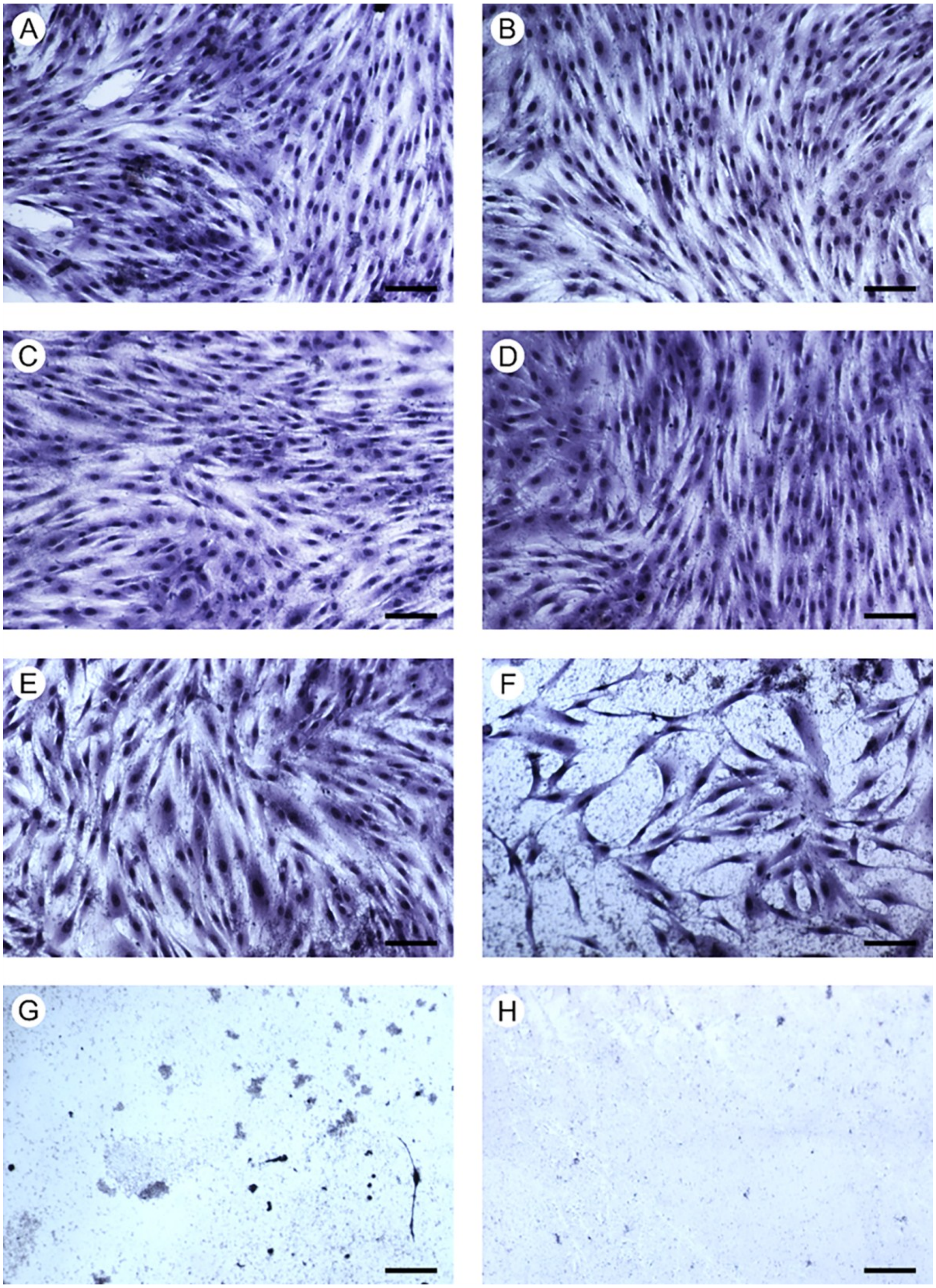

Supplementary Figure S5. Light microscopy images of the cellular morphology of HGF cells treated with AgNPs at 96 h (a) 0.0225 (b) 0.05 (c) 0.225 (d) 0.5 (e) 2.25 (f) 5 (g) 22.5 (h) 50 µg/mL. Scale bar = 100 µm.

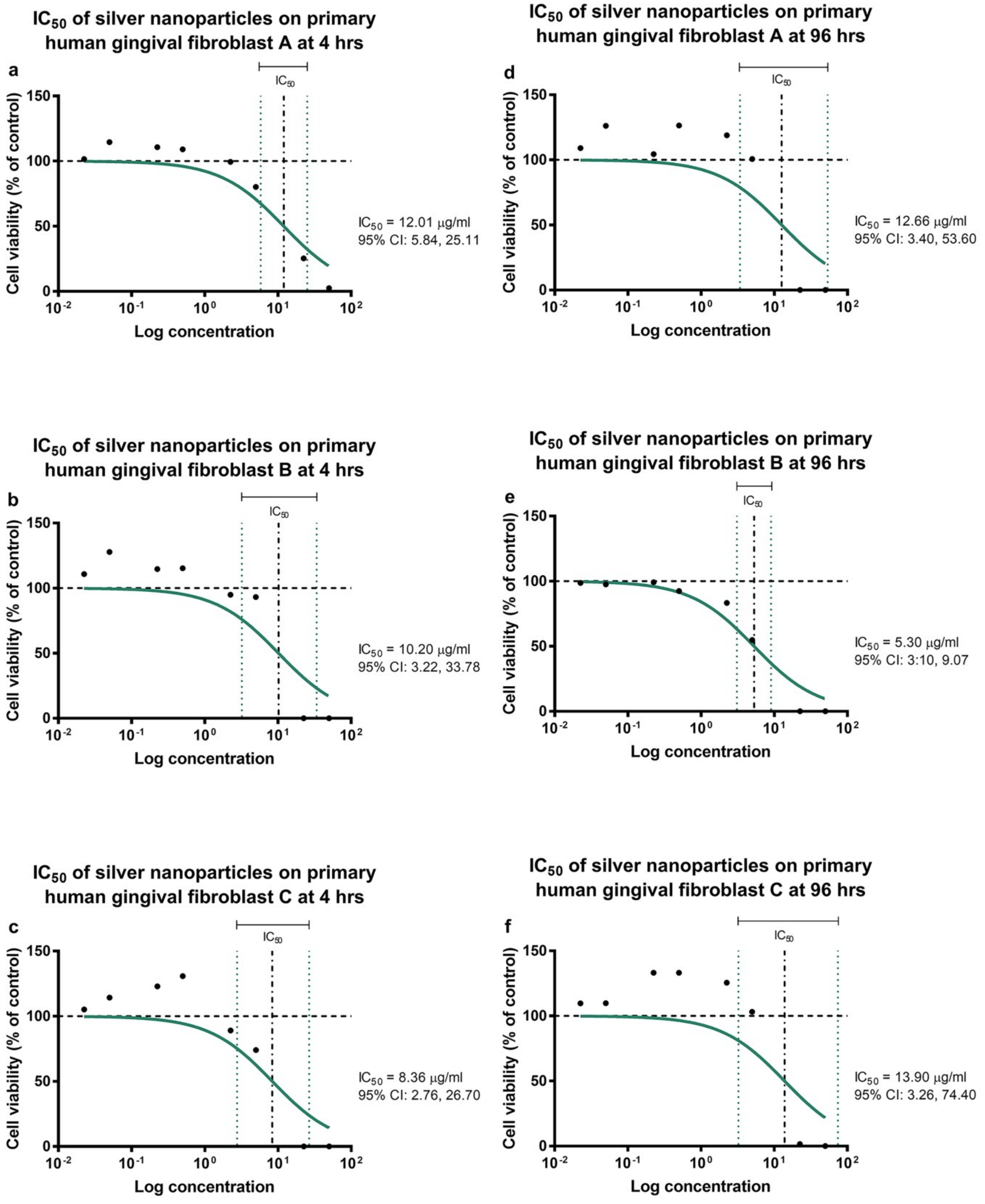

Supplementary Figure S6. Half maximal inhibitory concentration (IC₅₀) of AgNPs on HGF-1 at (a) 4 h (b) 96 h, HGF-2 at (c) 4 h (d) 96 h, HGF-3 at (e) 4 h (f) 96 h.
